# The Potential of Computer Vision-Based Marker-Less Human Motion Analysis for Rehabilitation

**DOI:** 10.1177/11795727211022330

**Published:** 2021-07-05

**Authors:** Thomas Hellsten, Jonny Karlsson, Muhammed Shamsuzzaman, Göran Pulkkis

**Affiliations:** 1 Department of Health and Wellbeing, Arcada University of Applied Sciences, Helsinki, Finland; 2 Department of Business Administration and Analytics, Arcada University of Applied Sciences, Helsinki, Finland

**Keywords:** Computer vision, marker-less, motion analysis, telerehabilitation

## Abstract

**Background:**

Several factors, including the aging population and the recent corona pandemic, have increased the need for cost effective, easy-to-use and reliable telerehabilitation services. Computer vision-based marker-less human pose estimation is a promising variant of telerehabilitation and is currently an intensive research topic. It has attracted significant interest for detailed motion analysis, as it does not need arrangement of external fiducials while capturing motion data from images. This is promising for rehabilitation applications, as they enable analysis and supervision of clients’ exercises and reduce clients’ need for visiting physiotherapists in person. However, development of a marker-less motion analysis system with precise accuracy for joint identification, joint angle measurements and advanced motion analysis is an open challenge.

**Objectives:**

The main objective of this paper is to provide a critical overview of recent computer vision-based marker-less human pose estimation systems and their applicability for rehabilitation application. An overview of some existing marker-less rehabilitation applications is also provided.

**Methods:**

This paper presents a critical review of recent computer vision-based marker-less human pose estimation systems with focus on their provided joint localization accuracy in comparison to physiotherapy requirements and ease of use. The accuracy, in terms of the capability to measure the knee angle, is analysed using simulation.

**Results:**

Current pose estimation systems use 2D, 3D, multiple and single view-based techniques. The most promising techniques from a physiotherapy point of view are 3D marker-less pose estimation based on a single view as these can perform advanced motion analysis of the human body while only requiring a single camera and a computing device. Preliminary simulations reveal that some proposed systems already provide a sufficient accuracy for 2D joint angle estimations.

**Conclusions:**

Even though test results of different applications for some proposed techniques are promising, more rigour testing is required for validating their accuracy before they can be widely adopted in advanced rehabilitation applications.

## Introduction

The need for low cost, easy and equally achieved rehabilitation services for the population has led to a search for new ways to implement them. The recent corona pandemic has further forced health care and welfare sectors to find new digital alternatives for treating their clients. A possible availability improvement in rehabilitation is through telerehabilitation based on technologies like Computer Vision (CV). A significant telerehabilitation advantage is the unnecessity of clients’ physical travel to the therapist. Hence, they save time and costs, they can decide themselves when they do the therapeutic exercises, and it is easier to integrate the exercise into the daily activities. The benefit of CV based telerehabilitation is that the only technical equipment needed is a camera/cameras attached to a computing device for performing human motion analysis.

Tracking and analysis of human movement has been an intensive research topic for decades. Traditional CV based motion analysis uses marker-based approaches. However, the requirements of a controlled environment and very precisely calibrated equipment mean high acquisition costs.^1^ Furthermore, marker-based systems typically require attachment of physical markers to strategic body points for automatic kinematic data collection. This limitation makes routine use of motion analysis systems impractical, as it requires significant technical preparations prior to rehabilitation performance.

A significantly more practical and easy-to-use solution is CV based marker-less motion analysis. In this solution, a client only needs a computing device with one or more cameras attached for performing therapeutic exercises guided by the application. Literature sources propose several generic marker-less motion analysis systems, typically utilizing emerging CV and machine learning-based techniques. These techniques are systems finding joint coordinates in a 2D space and systems localizing joints in 3D space, typically multiple cameras but also single-camera 3D motion analysis systems are proposed. Considering practicality and user-friendliness, the optimal case, from a physiotherapy point of view, would be a single camera system capable of accurately finding the 3D coordinates of all joints. This would enable analysis of advanced joint movements, such as hip and shoulders analysis in functional movements like walking and squatting but also simple movements as arm or leg stretching and bending.

However, CV based marker-less 3D pose estimation from a single image view is a challenge. Development of such systems has hitherto mostly been for the entertainment domain, but some systems exist also for sports and rehabilitation. A general issue of current marker-less motion tracking systems is the difficulty to achieve sufficient accuracy.^2,3^ Marker-less systems are therefore not widely used within rehabilitation.

This paper provides an overview of recent CV based marker-less human motion analysis systems, validates the accuracy and practicality of these systems from a physiotherapy point of view and discusses their usability for rehabilitation both generally and with a practical example. The main purpose is to provide a state-of-the-art review of marker-less human pose estimation and their suitability for rehabilitation aids and to evaluate the need for future research.

The structure of the rest of the paper is as follows. Section 2 presents related research. Section 3 discusses requirements of CV based motion analysis for physiotherapy needs. Section 4 provides an overview of recent generic CV based marker-less human pose estimation systems, their performance and accuracy. Section 5 presents some rehabilitation aids using CV-based human motion analysis. Section 6 proposes some future research directions. Section 7 evaluates critically the suitability of current CV based marker-less systems and presents some concluding remarks.

## Related Research

Human motion analysis for rehabilitation has been an active research topic for more the 30 years.^4^ Several reviews on CV based human motion analysis have already been published. Moeslund et al^5^ and Poppe^6^ survey vision-based human motion analysis. The thorough survey by Zhou and Hu^4^ on human motion tracking for rehabilitation covers non-visual tracking, visual marker based tracking and visual marker-free based tracking for both 2D and 3D approaches. Holte et al^7^ present developments in human pose estimation and activity recognition from videos. Yang et al^8^ present an overview of marker-less motion capture systems for person tracking. Colyer et al^2^ and Mündermann et al^9^ survey CV based motion analysis evolution towards a marker-less system. Colyer et al^2^ point out that the widespread manual cine film camera recording digitization prior to digital technologies did not necessarily require the attachment of markers. The survey also presents some` commercial computer vision based marker-less motion analysis systems and surveys recently published studies on the accuracy of computer vision based marker-less human motion analysis in comparison with other human motion analysis systems. The systematic review by Webster and Celik^10^ on Kinect camera system^11^ applications includes stroke rehabilitation.

## The Requirements on Computer Vision-Based Motion Analysis from a Physiotherapist Point of View

‘Telerehabilitation refers to the delivery of rehabilitation and habilitation services via information and communication technologies (ICT)’.^12^ Client and therapist are differently located and communicate with ICT technologies in a rehabilitation process.^13^ However, the process should resemble traditional rehabilitation where the client and the physiotherapist work in the same room. The process thus consists of a physiotherapy intervention where the interaction between the physiotherapist and the client starts from the physiotherapist’s interview and a clinical analyse ending up in a physiotherapeutic diagnose with a set goal and an intervention plan.^14^

The digital intervention used in CV for self-managing therapeutic exercise should consist of 3 central tools: catching the exercise in real-time, understand the exercise and to evaluate the performance. On the base of these components, the application should be able to give personalized feedback.^15^ In order to be effective the web-based platform used in CV assisted rehabilitation must ensure correct exercise performance of the client and automatic detection of wrong executing. When a client performs an exercise in front of a machine replacing a physiotherapist, then the client can perform movements wrongly. Especially after a surgery, this can be harmful, and the absence of a therapist can affect the motivation and slow down the recovery process.^16^ A client’s motivation to do the exercise is important for the rehabilitation process. Digital intervention capturing the exercise and providing real-time feedback can be one solution to support the process.^15^ Useful motion analysis software should include a professional system to analyse movements like a therapist.^16^

To integrate CV in clinical rehabilitation, the assessment must be valid and reliable to be objective.^17^ CV use in rehabilitation assist the clients and physiotherapists assessment of the movements that the client is performing. The expected CV application output is the most important thing for enabling the application to measure and analyse movements correctly. When this is the case, the application is implementable in the rehabilitation process.

In clinical work, physiotherapists use universal goniometers to measure their clients’ joint angles to follow up the rehabilitation process. Valid goniometric measurements are important data for physiotherapists’ clinical decision-making.^18^ Some variation in a goniometric joint angle measurement can emerge, if the physiotherapist has improper placement of its fulcrum over the centre of rotation of the joint or wrong anatomic structures.^19^ Melián-Ortiz et al^20^ have shown, that the variation can be 1° to 7° from the actual degree in manual joint measurements with a universal goniometer. To minimize the variation, physiotherapists should follow standard written principals.

## Computer Vision-Based Marker-Less Human Motion Analysis

Colyer et al^2^ consider that marker-less motion capture consists of the following 4 components:

1. The used camera systems.
2. The human body model.
3. Image features for motion capture.
4. The algorithms determining shape, pose and location of the body model.

Their review classifies used camera systems into depth-sensing camera systems and other camera systems, points out that marker-based and marker-less body motion capture use similar body model, discusses the use of some image feature (silhouettes, visual hulls and colour models) in human motion capture, and classifies algorithms for body model analysis into generative and discriminative algorithms. A generative algorithm fits the body model of a person to extracted image information while a discriminative algorithm requires training using machine learning to be able to discover mappings directly from image features. This section presents (1) camera systems, (2) human pose estimation which covers body models, image features motion capture and algorithms determining body model parameters and (3) performance and accuracy of CV based marker-less human motion analysis.

### Camera systems

Marker-less human motion capture can use ordinary cameras where each image pixel has colour and brightness and/or depth-sensing cameras where each image pixel describes the distance from a space point to the camera.^2^ Depth cameras are narrow-baseline binocular-stereo cameras, for example Stereolab’s Zed Camera,^21^ and ‘active’ cameras which depth-sense from reflection of emitted light into the observed scene, for example Micosoft’s Kinect camera.^11^ Active depth cameras, also called RGB-D cameras, also capture image pixel colour. These camera systems use either structured light to sense depth from known patterns projected onto the illuminated scene or time-of flight to measure the reflection time of a light pulse.^22^

### Human pose estimation

CV based human pose estimation systems are 2D and 3D. A 2D pose estimation estimates (*x, y*) coordinates of each joint in an image. A 3D pose estimation finds the corresponding (*x, y, z*) coordinates. From a physiotherapy point of view, 3D systems add more value as most human joints are movable in multiple directions. Accurate motion analysis therefore requires joint detection and localization in a 3D coordinate system. Recent human pose estimation systems use deep learning, a branch of machine learning, which maps the relation among the features directly on diversely represented data. It learns hierarchical information from the data and afterward weights hierarchies to compute the predicted output. Due to its hierarchical information gathering property and high approximation capacity, deep learning is modern machine learning.^23^ Yang et al^24^ apply deep learning to a low-resolution single image to obtain a high-resolution image version. Yang and Ramanan^25^ present a deep learning based human pose estimation system.

#### A 2D pose estimation

Since 2D systems are often the base of 3D pose estimation, this subsection presents an overview of existing 2D pose estimation systems. There are several different proposed approaches to 2D pose estimation in recent years. Initial proposals were handcrafted features such as histogram of oriented gradients (HOG)^26^ and Edgelet.^27^ However, these proposals suffer from insufficient accurately in detecting body parts and hence deep learning methods are currently evolving.^28^ The strength of deep learning, in contrast to handcrafted feature-based solutions, is its capability of extracting sufficient features from metadata.

The general techniques used for 2D pose estimation of a single person are direct regression and heat-map based techniques.^28^ In direct regression, key points from the body are directly regressed in 1 single step. DeepPose,^29^ a method for human pose estimation based on deep neural networks (DNN), is one of the first human pose estimation solutions using deep learning and direct regression. The model architecture is to a major extent convolutional and uses fully connected output layers for predicting joint coordinates directly as numerical values.

**Figure 1.**
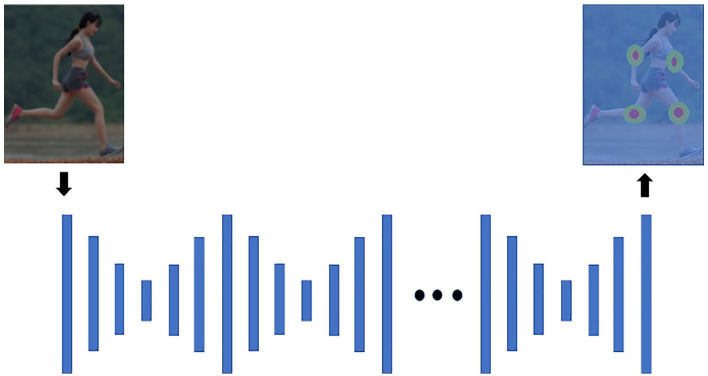
A visualization of the repetitive stacked hourglass approach.

A heat-map based solution predicts the probability of a joint occurring in each pixel. Common approaches for 2D pose estimation are the generation of joint heat-maps, such as the stacked hourglass approach.^30^ In this approach, processing and extracting features down to a low resolution uses convolutional and max pooling layers. Once reaching the lowest resolution, a top-down sampling sequence combines features across scales. Repeating this process of bottom-up and top-down sampling several times enables the network to reach sufficient output resolution (see Figure 1). Thereafter 2 consecutive rounds of 1x1 convolutions produce the final network predictions. The final output is a set of heat-maps where each heat-map represents a prediction of the probability for a joint being present in each pixel.

OpenPose^31^ is a multi-person pose estimation system that has formed the base for several recent pose estimation solutions. The main reason for its popularity is its capability of detecting up to 25 joints for each person thus making it useful for performing advanced human motion analysis. In OpenPose a convolutional neural network uses a multiple-stage classifier wherein each stage the result of the previous stage is improved. In the first stage, the input is an original image for prediction of possible locations of each key point in the image. The output is a confidence map equivalent to a heat-map. Each subsequent stage takes the image data together with the confidence map produced at the previous stage for improving the accuracy of the heat-map for each stage. Introduction of the idea of part affinity maps (PAF) within OpenPose trains the model to associate body parts with specific persons in an image. As a result, OpenPose is a bottom-up solution providing accurate real-time pose estimation regardless of the number of people in an image.

#### A 3D pose estimation

This subsection presents an overview of the most recent generic and application-specific marker-less multi-view 3D pose estimation systems. Pre-deep learning is a common approach to 3D pose estimation, especially used for hand from a colour image considering discriminative and generative methods. Unfortunately, the performance of these methods insufficient due to the dependencies on different factors such as prior knowledge of the image background, careful initialization etc.^32^

##### Systems based on image streams from multiple cameras

**Figure 2.**
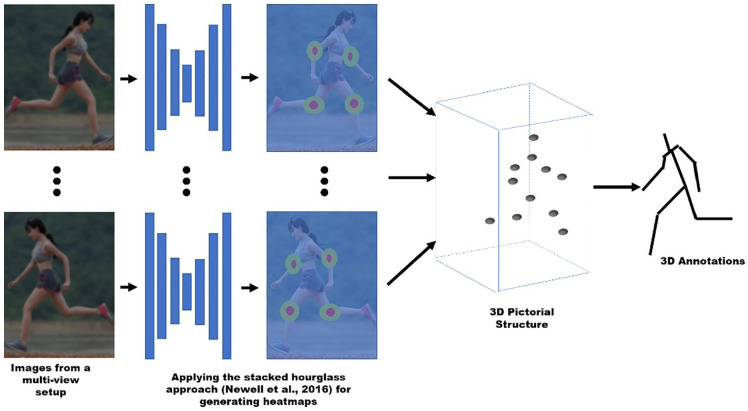
Components of a geometry-driven approach to human pose estimation.

For achieving an accurate joint estimation in a 3D space, many proposals focus on environments with multiple camera streams taken from different angles. Pavlakos et al^33^ propose a geometry-driven approach automatically collecting annotations for human pose prediction tasks. Figure 2 shows the different components of this approach.

The initial component applies the stacked hourglass approach^30^ for generating 2D heat-maps for each joint in each view as outputs. Combining each heat-map using a 3D pictorial structure model performs 3D pose estimation.^34^ Finally, further examination of the 3D pose estimate determines reliable joints for use as annotations.

**Figure 3.**
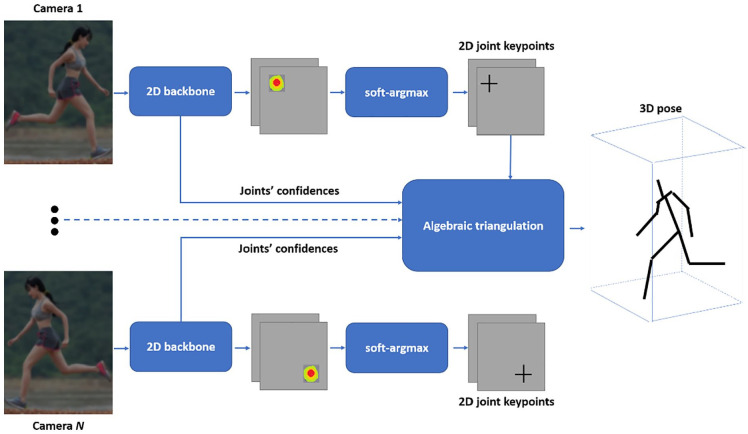
A proposed multi-view 3D poses estimation system.

Iskakov et al^35^ propose a multi-view 3D pose estimation system based on the learnable triangulation of human pose. The input of the system, visually outlined in Figure 3, is a set of images captured from N cameras with known parameters.

Off-the-shelf 2D human detectors crop each captured frame. The cropped images are used as inputs to a deep convolutional neural network backbone based on a proposed architecture for production of joint heat-maps and joints’ confidences.^36^ Then, application of a softmax-argmax function calculates the 2D joint positions as the center of mass of the corresponding heat-maps. Finally, derivation of the 3D positions of the joints from the corresponding 2D joint estimates from multiple views uses a proposed linear algebraic triangulation approach.^37^

Shere et al^38^ propose a multi-person 3D skeleton pose estimation system using a pair of 360° cameras. Initially, 2 video recorders capture 360° sequences of an environment consisting of multiple persons. Tracking of each person occurs across the 2D image sequence. OpenPose estimates joint locations from these tracks.^31^ In this case OpenPose provides for each frame a set of 2D joint coordinates, where each joint has an index and a number indicating the camera providing the frame. Assume, for instance, that the same joint appears in 2 different camera frames. A triangulation method estimates the 3D coordinate for that joint. Next follows bone length estimation by measuring the distance between each 3D joint coordinate. Finally follows gradient descent optimization using Ceres^39^ for finding a skeleton pose fitting the joint estimates in the most optimal way. As a result, the solution can track humans from a multi-person image and provides a skeletal pose of each person.

##### Systems based on an image stream from a single camera

This section is an overview of marker-less systems using a single camera/monocular video stream. Huang et al^40^ focus on multi-view pose, but claim that their method is applicable to monocular video sequences without large modifications.

There are many proposed approaches for detecting 3D poses of the human body from a single image with and without background exclusion. For example, SCAPE is an important approach to 3D pose detection and estimation. It is among the first parametric models fitted to the ground truth image to estimate a high-quality 3D pose.^41^

Accessibility of large datasets of 3D shapes and the deep learning performance advancement facilitated 3D reconstruction from a single image.^42^ Zimmermann and Brox^43^ used deep learning to estimate 3D hand pose from single images where a foremost part of training datasets was synthetic. Their project used through concatenation 3 networks for hand segmentation and successive 2D and 3D joint prediction. However, Mueller et al^44^ found a weakness in the project due to many synthetic datasets. Alternatively, they proposed for image translation a Cycle-GAN technique, which translated synthetic images into real looking ones. Later, translated data for 2D and 3D joints prediction trained a regression. Furthermore, a convolution neural network (CNN) and optimization produced a better 2D joint prediction result.^45^

Many recent works estimate a substantial 3D reconstruction of the human body, but mostly inaccurately predict the 3D pose of a person. These works follow an end-to-end model, which predicts 3D joint locations, regress 3D heat-maps and classifies images based on their pose class.^46^ Pavlakos et al^3^ estimate 3D human pose and shape from a single colour image. Bogo et al^47^ describe automatic estimation of 3D human pose and shape from a single image.

**Figure 4.**
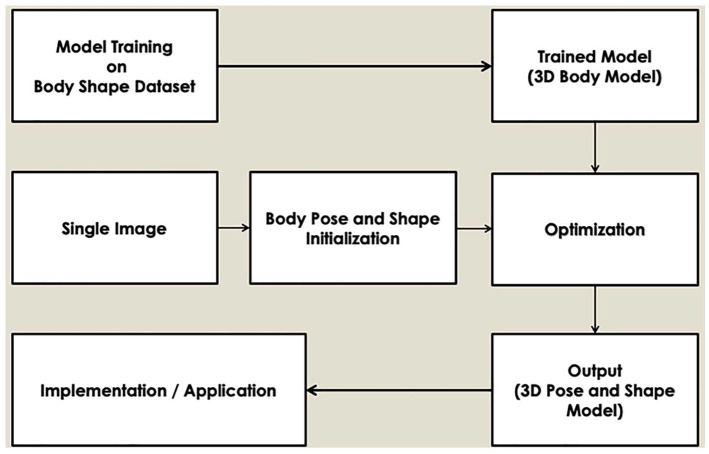
A framework of using single image to infer the shape and pose of a human body.

Kanazawa et al^48^ present end-to-end recovery of human shape and pose. Their approach uses 2-stage and direct estimation. Prediction of the 2D joint locations used firstly 2 stage methods, 2D pose detectors or ground truth 2D pose. Thereafter prediction of 3D joint locations from 2D joints locations used a regression or model fitting. Given a single image and minimal user input, Figure 4 shows computation of an initial pose, light direction, shape and segmentation.

Furthermore, also direct prediction approaches, which are discriminative and keep the optimization objective unclear during implicating the information, can estimate 3D pose and shape. For example, using these approaches a convolution network has detected 91 human body landmarks. A random forest estimated the 3D body shape from these landmarks. Training use of these landmarks still requires the positions of the body shapes.^3^

### Performance and accuracy

**Figure 5.**
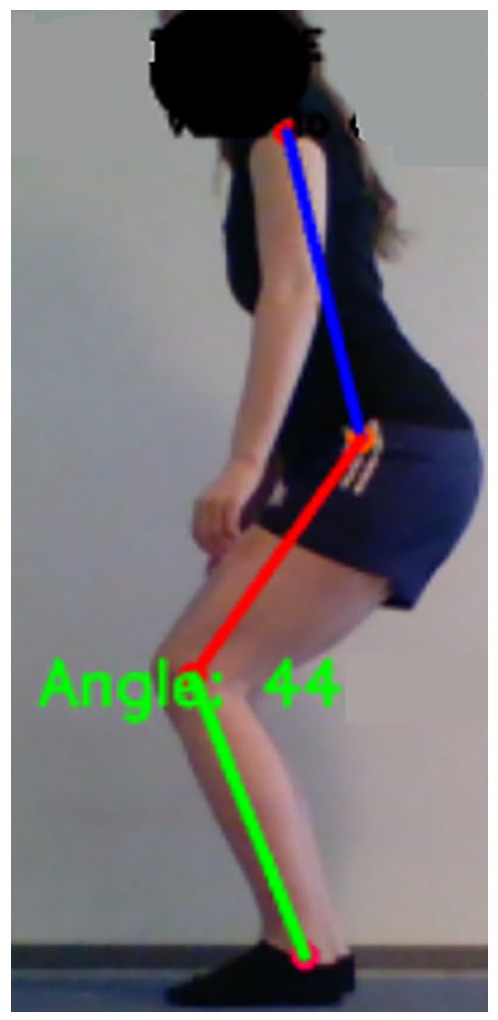
An illustration of a marker-less CV application measuring the knee joint angle.

**Figure 6.**
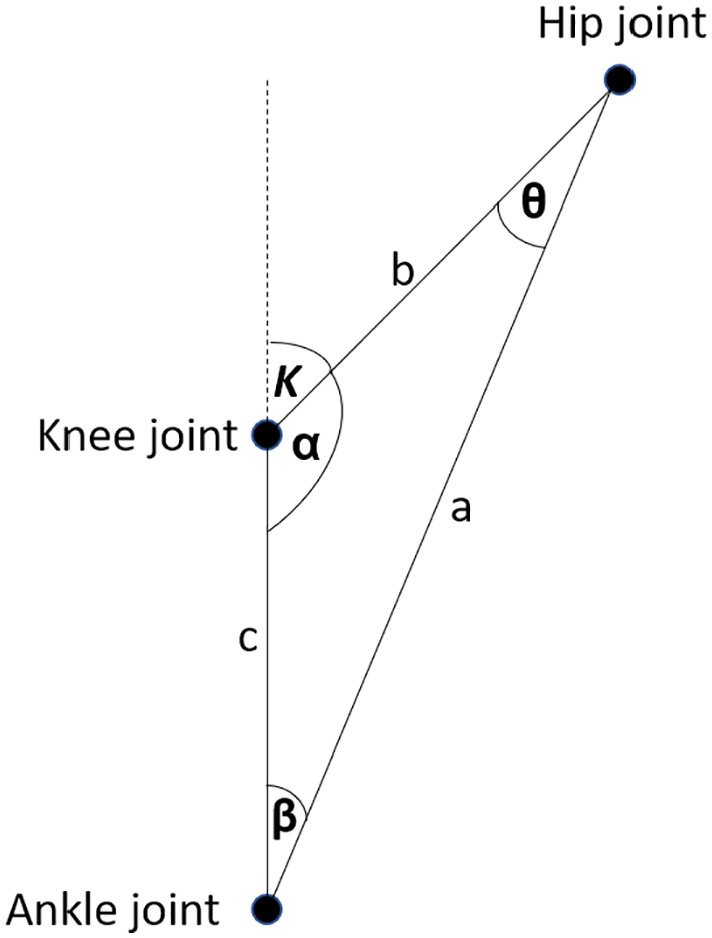
After estimating the coordinates of hip, knee and ankle joints, the knee angle *K* estimation applies the law of cosines.

A critical issue related to all proposed marker-less human pose estimation systems is their accuracy level. Colyer et al^2^ and Pavlakos et al^3^ stated that due to accuracy issues marker-less systems are not yet widely used within biomechanics. This section analyses the accuracy of some recent techniques and their theoretical suitability for rehabilitation aids. A simple example is measurement of the angle of the knee joint, see Figure 5. In this measurement, the client is either lying down or standing up with his/her side directed towards the camera. First, the application estimates the coordinates of the hip, knee and ankle joints. These coordinates form a triangle, as shown in Figure 6. Estimation of all corner angles applies the law of cosines after calculating the Euclidean distances between all coordinates. The angle of interest is *K* in Figure 6 and its calculation uses the following equation:

$$K=180-a\cos\left(\frac{a^{2}-b^{2}-c^{2}}{-2ac}\right)$$

The precision of the estimated knee joint coordinates is crucial for estimating a knee angle with sufficient accuracy. As discussed in section 2, the maximum tolerable error margin in joint angle measurement for physiotherapy purposes is ±5°. PCK (Percentage of Correct Key points) or its modified variant PCKh often represents joint localization performance of pose estimation proposals. PCKh indicates a percentage value for the probability of correctly detecting a specific joint coordinate. The distance between the estimated and the actual joint coordinate is within the range of the head length for a correctly detected joint. A typical threshold value for PCKh is 0.5 denoted as PCKh at 0.5, meaning 50% of the head length. Cao et al^31^ present results showing that OpenPose can accurately detect head, shoulder, elbow, wrist, hip, knee and ankle joints with a mean probability of up to 80% when PCKh at 0.5 is applied.

**Figure 7.**
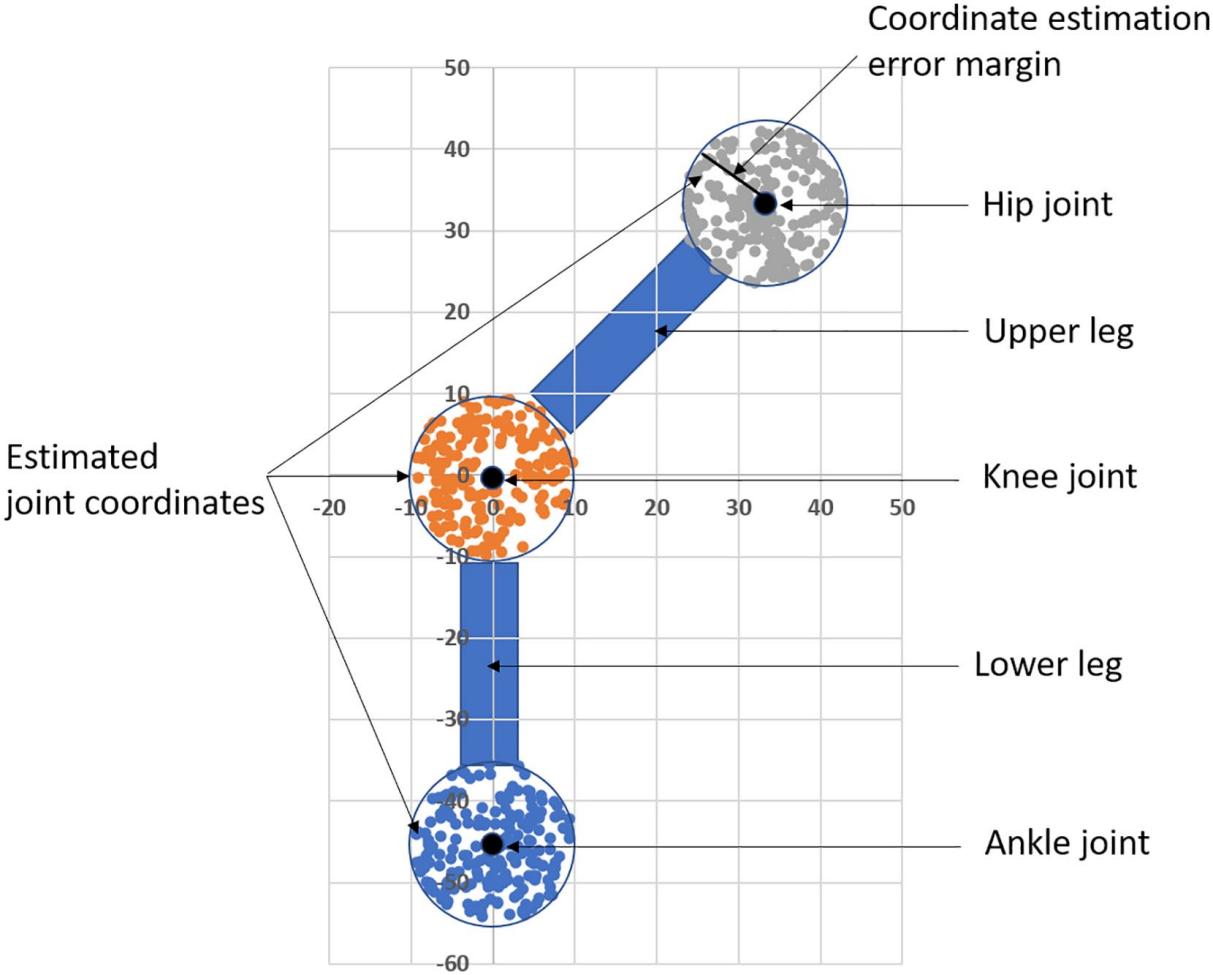
An illustration of a simulation tool calculating the level of accuracy for knee angle measurement for different error margins of joint coordinate estimations.

However, PCKh at 0.5 is not a sufficient threshold for joint angle measurements. Simulation demonstrates this as is shown in Figure 7. The black dots are the actual coordinates of the hip, knee and ankle joints while the coloured dots are estimations. An error margin for the estimated joint coordinates is the maximum distance from the actual joint in a circular area. The simulation generates uniformly random coordinates for all joints within the specified error margins to calculate the knee angle from random joint coordinates. After N repetitions of this procedure, dividing the number of knee angle measurements within the tolerable margin with the total number of measurements gives the probability of estimating the knee angle within the tolerable error margin (±5°).

For an average male, the upper leg length, that is the distance between the hip and knee joint, is 46 cm. The lower leg length is 45 cm and the head length is approximately 20 cm. Application of PCKh at 0.5 means in practice, that the error margin for the joint coordinate estimate is 10 cm.

Assuming the real knee angle to 45°, then simulation with these parameters and N = 1000 shows, that the probability for measuring the knee angle within a ±5° margin is only 25%. To guarantee a maximum error margin of ±5°, when measuring the knee angle in this particular example, the error margin in estimating the joint coordinates can be at most 2.5 cm, which approximately corresponds to PCKh at 0.1.

The proposals of Slembrouck et al^49^ and Gu et al^50^ use OpenPose to detect 2D coordinates of human joints. Gu et al^50^ analyse the performance and accuracy of OpenPose in computing lower limb angles from tracking joint coordinates of walking adults with a single cell phone RGB 30 Hz camera. A state-of-the-art commercial multi-camera system validated the computed lower limb angle values. The angle error was 10° or less in most tracking frames, an accuracy comparable with the accuracy of a marker-based depth camera system.^51^ Slembrouck et al^49^ use triangulation. A 2D joint coordinates detected by OpenPose are further progressed into 3D joint coordinates by applying OpenPose on camera images from multiple angles before least squares triangulation. The authors claim that their system can track the pose of multiple persons in real-time with a frame rate between 20 and 25 fps and that lower body joint coordinates are detected with a standard deviation between 9.6 and 23.7 mm. In the above-mentioned example, this means that their system could detect human joints with a tolerable accuracy for knee measurement purposes with a probability of approximately 70%.

Another proposal uses OpenPose with multiple synchronized video cameras to compare marker-less motion capture accuracy with optical marker-based motion capture accuracy for 2 tested participants’ walking, countermovement jumping and ball throwing.^52^ Mean absolute errors (MAE) measured test participants’ corresponding joint position differences. For approximately 47% of all measurement MAE < 20 mm and for 80% of all measurement MAE < 30 mm. The measurements thus indicated that 3D pose estimation with marker-less motion capture was a correct reproduction of the test participants’ movements. A rough estimate is therefore that the knee angle measurement has approximately the same accuracy as the proposal by Nakano et al.^52^

Schmitz et al^53^ compare the accuracy of single camera marker-less motion capture with a ten-camera marker-based motion analysis system for 6 different postures of a jig simulating a human leg. A digital inclinometer with an accuracy of 0.1° measured the abduction-adduction angles, which both the marker-based system and the marker-less system calculated as the mean of 30 frames. The deviation from the inclinometer measurements was less than 0.5° for both systems. The marker-based system estimated abduction more accurately and the marker-less system was more accurate for adduction, but the difference was ±0.5° or less. Statistical comparison of the accuracy of both systems used a t-test with a significance level of 0.05.

## Computer Vision-Based Marker-Less Rehabilitation Aids

With a well working CV based marker-less approach the motion analysis could take place, for example, in the client’s home.^2^ In normal face-to-face clinical cases, the travel cost that is necessary for the session or the chance for therapy can be the main barriers for a client to get help.^54^ Some CV based real-time monitoring aids for rehabilitation have already been proposed and implemented. This section presents some recent examples.

Balance is a central task for people and especially for elderly. It is important to be able to identify fallers, because the injuries that becomes when a person fall can be severe. Most of the injuries are mild, but 5% to 10% of the injuries are severe for people that are older than 65 years.^55^ To be able to identify possible fallers, Nalci et al^56^ analysed with the help of marker-less CV standing on one foot with eyes open and closed. Thereafter they compared the results with a golden standard balance board that analysed the sway. An experimental setup used a Dynamic Vision Sensor camera to capture pixel-level illumination changes of motion. The results had a high correlation and shows that CV can in the future be a possible equipment used in balance measuring in rehabilitation to identify possible fallers.

Homebased exercises supervised by a therapist are one of the most important treatment in the recovery phase in several diagnosis like in osteoarthritis (OA) or stoke. The goal of home based exercises can be to decrease pain in the joint and get better functionality but also to lower the costs for the therapy.^57-59^ CV-aided systems that can capture exercises and give feedback can be a key to an effective and successful rehabilitation process. Without feedback, therapeutic programs are difficult to personalize and motivate the client to do the exercises.^15^ There is also a possibility that if the clients do not get feedback they do the exercises wrongly. Especially after surgery, this can be harmful and slow down the recovery process.^16^ Dorado et al^60^ present a developed easy-to-use CV system called ArthriKin, which offers a possibility to interact directly with a therapist to make a home based exercise program efficient. Baptista et al^59^ have established a home-based training system for stroke patients. Their system uses 2 linked applications for the therapist and the client. The patient side application (Kinect) gives real-time and visual feedback but also reports how the client preforms the therapeutic exercise. There have also been developed CV models to track objects to assist stroke patients, which try to reach for and grasp objects with the aid of a robotic device. Rehabilitation of patients with an injured arm or wrist has likewise used CV.^61^ A web camera records a cuboid object and software calculates the object’s position in real-time when the patient tries to move the object to match the position of an already present virtual object in a virtual 3D space.

Salisbury et al^62^ demonstrated with a smartphone camera real-time measurements of a patient’s vestibular rehabilitation therapy. Vestibular rehabilitation is used for example patients with symptoms of dizziness and for elderly fall prevention. During Vojta therapy,^63^ body movements of patients having motor disabilities have been monitored and analysed in real-time with CV-based experimental methods.^64^ A CV-based action identification system for upper extremity rehabilitation in patients’ home environments has been proposed.^65^ The proposed system captures sequences of colour images with colour and depth of a patient’s upper extremity actions for identification of movements. The image sequence with a rate up to 125 images/s is processed and analysed to distinguish between correct and wrong rehabilitation actions in action training.

**Figure 8.**
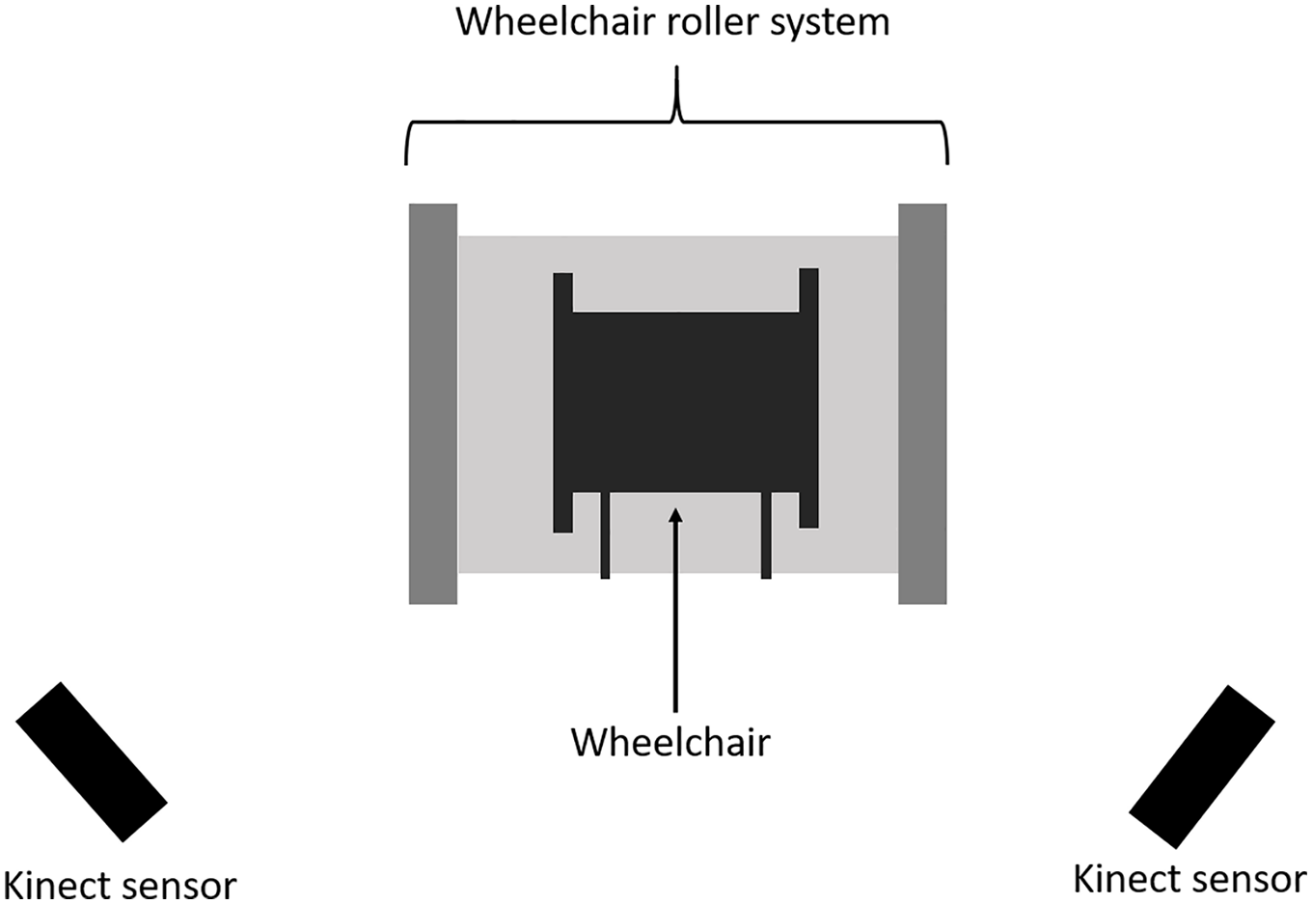
A marker-less system for analysing manual wheelchair propulsion.^66^

Rammer et al^66^ propose a system for marker-less motion analysis of manual wheelchair propulsion. Their system requires a minimum of two Microsoft Kinect sensors (hardware devices with camera and microphone) for capturing motion data. The motion analysis focuses on the upper extremity kinematics during wheelchair propulsion. The system utilizes OpenSim,^67^ an open-source software platform for biomechanical modelling, simulation and analysis. The wheelchair is located in a stationary wheelchair propulsion roller and the Kinect sensors are on each side of the wheelchair, see Figure 8.

**Figure 9.**
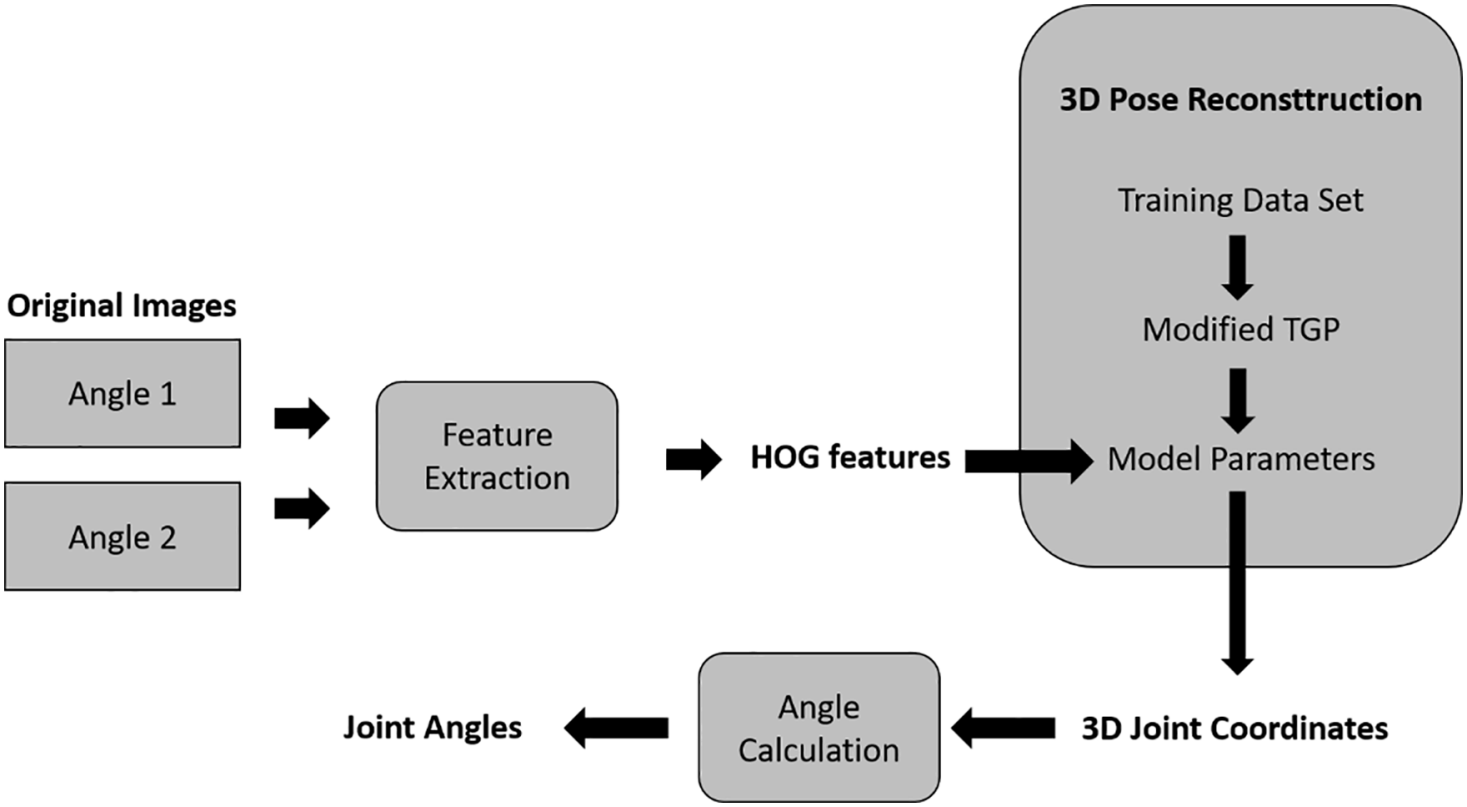
A CV based marker-less motion capture method to assess 3D joint kinematics of symmetrical lifting tasks.^68^

Mehrizi et al^68^ present the development and validation of a CV-based marker-less motion capture method to assess 3D joint kinematics of symmetrical lifting tasks. A new CV based method is proposed for image feature extraction and calculating the joints kinematics without the need of physical markers. Figure 9 shows a visual overview of the proposed method. In short, the method works as follows. Two optical cameras capture video images from 2 different angles. Thereafter, a technique called Histogram of Oriented Gradients (HOG) detects objects, which in this case means detection of the human body. The next step is a reconstruction of the 3D pose of the body for each video frame using a modified implementation of the Twin Gaussian Process (TGP). The output of this process is a set of the 3D coordinates of 45 virtual markers. Based on these markers, the joint angels are calculable for analysing the lifting task.

## Future Research Directions

Previous sections have presented several published proposals of marker-less CV based human pose estimation techniques. From a usability point of view, most presented proposals are promising for physiotherapy applications, as they only require a computing device together with one or more cameras. Many techniques already enable estimation of the 2D coordinates of human joints using only one camera and provide the potential for easy-to-use applications analysing simple mobility of some joints, such as knee and elbow. However, analysis of the mobility and functionality of more advanced joints, such as hip, wrists and shoulders require 3D pose estimation. Typical 3D pose estimation systems require multiple cameras, which is impractical from a usability point of view since application execution requires a complex camera setup. Some research papers propose single camera 3D pose estimation systems. These are the most promising for physiotherapy applications as they are easy to use (no complex camera setup) and capable of detecting the 3D coordinates of human joints.

A preliminary conclusion based on our literature review is that some proposals for human pose estimation already provide sufficient accuracy for physiotherapy needs. Accuracy differences in comparison with marker-based systems are negligible. However, implementation of existing solutions and testing these solutions for physiotherapy purposes is necessary before any reliable statement on their level of accuracy in comparison to each other is possible. Thus, the top priority for future research is to apply some of the most promising marker-less human pose estimation algorithms in physiotherapy applications for rigourous testing. The testing could start from 2D applications, for example knee and elbow angle measurements, and move on towards more complex 3D movement analysis applications of more advanced joints, such as hip and shoulders. Moreover, development of suitable machine learning-based calibration methods for CV based marker-less human motion analysis systems for rehabilitation applications requires future research.

## Conclusions

CV based marker-less pose estimation systems are attractive for rehabilitation aid applications as they can provide analysis and supervision of rehabilitation exercises for clients at home and thus reduce the need for physically meeting the physiotherapist. Marker-less motion analysis systems are easy-to-use, as they only require a camera/a set of cameras and a computing device. Before we can widely integrate CV into physiotherapy, however, the assessment and analysis of active movements must be valid and reliable. When a physiotherapist uses marker-less CV as assistive equipment in rehabilitation the therapist cannot manually or verbally instruct the movement that the client is performing, and the effect of the therapy, like for example the joint motion, cannot be measured by a physiotherapist. The most important issue is that the application can correctly and with sufficient accuracy measure and analyse movements a client is performing. The system should also be able to give real-time feedback so that the rehabilitation process is successful. Preliminary simulation results indicate that some recent CV based marker-less pose estimation systems already provide sufficient accuracy for joint detection and localization in joint angle estimations. However, implementation of existing solutions and rigourous testing of their accuracy is necessary and their accuracy in a range of real physiotherapy scenarios is necessary before they can be widely adopted in rehabilitation.
